# Correspondence

**Published:** 1884-03

**Authors:** J. S. Bryant

**Affiliations:** Marysville, Mo.


### CORRESPONDENCE.

MARYSVILLE, MO., January 11, 1884.

To the Editor of the DENTAL REGISTER:

DEAR SIR — I have recently made a discovery in working rubber that, I think, will be of great service to those who use rubber. Every one is familiar with the fact that in vulcanizing thick rubber it often becomes spongy, and is really spoiled, and no certain way has, as yet, been devised to obviate it. I have adopted a plan that completely overcomes the difficulty, and makes any thickness that may be needed, for the restoration of features, or anything of the kind. The method of accomplishing this is to take strips of vulcanized rubber of proper size, and thoroughly roughened with a rough file, or drill holes as in the preparation of a broken plate. Pack in these, in proper position, as many as the space will conveniently hold, so as to be entirely covered by the soft rubber, then vulcanize as usual. This will make strong, solid work, and without a possibility of sponginess.

Yours truly, J. S. BRYANT.

